# Supportive Care—A Missing Piece in the Current Global Efforts of Promoting Respectful Maternity Care

**DOI:** 10.3389/phrs.2024.1605597

**Published:** 2024-04-29

**Authors:** Waqas Hameed, Bushra Khan, Bilal Iqbal Avan

**Affiliations:** ^1^ Department of Community Health Sciences, Aga Khan University, Karachi, Sindh, Pakistan; ^2^ Department of Psychology, University of Karachi, Karachi, Sindh, Pakistan; ^3^ Department of Population Health, London School of Hygiene and Tropical Medicine, London, United Kingdom

**Keywords:** respectful maternity care, mistreatment, health system bottlenecks, human rights, disrespect and abuse, continuous support, psychosocial support, supportive care

## Introduction

Respectful and dignified maternity care is a fundamental right of every woman [1]. Over the past decade, numerous interventions have been tested to promote respectful maternity care in facility-based settings. However, these interventions seem to have neglected an important component of respectful maternity care (RMC)—that is “supportive care.”

In the context of RMC, supportive care (a.k.a psychosocial support) is “the provision of psychological and social strategies provided by the staff that aims to help a pregnant woman to tackle physical, mental and emotional challenges faced during intrapartum phase” [2]. The objectives of this commentary are: to highlight the significance of embedding “supportive care” more prominently for pregnant women and their companions in RMC interventions and programmes. Secondly, share findings of a critical review seminal RMC training manuals used in different part of the world, particularly in low-and middle-income countries (LMICs) to exam the operationalization of “supportive care” within their content. And third, and using reference of our recently published experimental work, Finally, share our recommendations on the way forward for the development of a holistic service delivery package for enabling the supportive and dignified maternity care.

## Significance of Supportive Care During Childbirth

A significant proportion of women appraise their experience of giving birth as traumatic [3]. Women suffering from psychological distress during labour are uniquely vulnerable to environmental influences such as unfamiliar personnel, medicalised procedures and other conditions [4]. In addition, women may face many socio-economic and health-related vulnerabilities, including, poor economic conditions, lack of social support, domestic violence, a history of either personal or familial mental illness and functional disability, all of which can influence a women’s childbirth experiences and outcomes [5]. While these vulnerabilities cannot be eliminated during intrapartum care, their effect can be alleviated through effective supportive care, leading to improved birthing outcomes. Women who receive supportive care during childbirth are: more likely have a shorter duration of labour, higher perceived control over birth, lower perceived labour pain, spontaneous vaginal birth, baby with a low five-minute Apgar score, and practice exclusively breastfeeding [4, 6].

## Supportive Care—A Component of RMC

Condescending attitude of service providers, discrimination, and inadequate psychosocial support during maternity care is prevalent worldwide, particularly in LMICs [7]. Responding to the alarming situation, World Health Organisation (WHO) revised the vision for improved quality of maternal and newborn care by incorporating elements of respect and dignity, effective communication, and emotional support as fundamental right of every women [8]. More recently, WHO published a comprehensive set of evidence-based recommendations to promote a positive user experience of intrapartum care [9], and integration of perinatal mental health into maternal and child health services [10].

“Supportive care” is now an integral part of WHO’s current framework [8] and policy recommendations [9, 10], and is also reflected in WHO’s definition of RMC [9]; in fact, lack of supportive care is identified as one of the types of mistreatment [7]. The inclusion of supportive care in recent guidelines is driven by a growing body of evidence on the positive effect of birthing outcomes described above [4, 11].

## Approach

Grounded in empirical evidence and WHO guidelines, several interventions have been developed and used over the past decade to promote RMC in facility-based settings [12]. We have critically reviewed training manuals identified through systematic review on RMC intervention [12] and were either available in the public domain or willingly shared with us by the lead author (see Supplementary Table S1). These manuals aimed to build the capacity of service providers on RMC. Additionally, we undertook a google search to find other publicly available training material.

We primarily looked at two things in the content: a) provider-focused information in light of the mistreatment framework proposed by Bohren and colleagues [7]; and b) health system relevance–what strategies were proposed in the training manual to operationalise RMC within the healthcare system. The mistreatment framework is based on a comprehensive mixed-method systematic review to conceptualize and measure types of mistreatment women may face during childbirth.

## Results From a Review of RMC Training Manuals

This study reviewed six RMC training manuals from various organizations (Population Council, MCHIP, Ethiopia Ministry of Health, Tanzania project) and the key findings of our review as summarised in Table 1.

**TABLE 1:** Respectful maternity care training manuals included in the review (Global, 2013-2017).

Region/country, year of study	Author	Duration of training	Provider-focused						System-focused
			Abuse	Inclusivity	Standard of care	Effective communication	Supportive care for patients	Supportive care for provider	Operationalization
Global, 2013	Maternal and Child Health Integrated Program	One-day	Y	Y	Y	Y	N	N	N
Tanzania, 2014	Hannah L. Ratcliffe	Two-Days	Y	Y	Y	Y	N	N	N
Kenya, 2014	Population Council	Three days	Y	Y	Y	Y	N	Y	N
Nigeria, 2017	Federal Ministry of Health	Three days	Y	Y	Y	Y	N	Y	N
Afghanistan, 2017	Federal Ministry of Health	Three days	Y	Y	Y	Y	N	Y	N
Ethiopia, 2017	Federal Ministry of Health	Six days	Y	Y	Y	Y	N	N	N

## Content

Provider-focused: almost all manuals addressed different forms of mistreatment and emphasized human rights and ethics in quality care. The Population Council manuals additionally focused on provider attitude transformation and included psychological debriefing. MCHIP offered a global perspective solely on RMC types. The Ethiopian manual emphasized “compassionate care” within an ethical framework. The Tanzanian project used the Bowser et al. framework and White Ribbon Alliance charter for RMC descriptions. Notably, none explicitly addressed supportive care provision.

System-focused: While all manuals utilized effective teaching methods like case studies and discussions, concerns existed regarding RMC integration within health systems. The Population Council manuals suggested monitoring committees and “Open Birth/Maternity Days” for transparency and community engagement. The Ethiopian manual proposed “compassionate leaders” to drive the RMC agenda. However, all needed a comprehensive strategic framework for action plan development.

### Conclusion

Despite WHO’s framework and recommendations around RMC, supportive care is not distinctly covered in the existing RMC training manuals used in different LMICs.

Therefore, we advocate for a more comprehensive approach to supportive care by integrating psychosocial support for pregnant women including mental, emotional, and social aspects. To effectively deliver psychosocial support, a systematic, a two pronged strategy is necessary: Provider-Level: capacity building of maternity staff in evidence-based psychosocial support strategies that enable them to provide dignified care to pregnant women and their companions. Health System-Level: process development of for the systematic delivery of comprehensive care for assessing patients’ psychosocial needs and socio-demographic and health-related vulnerabilities, providing targeted psychosocial support, and making referrals when necessary. Such an approach guarantees comprehensive tailored to the specific psychosocial context of each patient. This will ensure that maternity care is responsive and personalized, catering to the unique needs of every woman, without discrimination. By addressing both provider and health system levels, we can create a more supportive, effective, and equitable maternity care environment.
