# Supplementary material for: Supportive Care—A Missing Piece in the Current Global Efforts of Promoting Respectful Maternity Care
Source: Public Health Rev. 2024 Apr 29;45:1605597. doi: 10.3389/phrs.2024.1605597 (PMC11089184; doi:10.3389/phrs.2024.1605597)
Supplement: Supplementary file 1 [file Table1.DOCX]

**Supplementary file**

**Sup. Table 1: Detailed references of RMC Training Manuals included in the review (Global, 2013-2017)**

| **Region or country, year of study** | **Author** | **Reference** |
| --- | --- | --- |
| Global, 2013 | Maternal and Child Health Integrated Program | Respectful Maternity Care Workshop: Learning Resource Package \| Quality of Care Network. Baltimore: Jhpiego, 2013 https://www.qualityofcarenetwork.org/knowledge-library/respectful-maternity-care-workshop-learning-resource-package (accessed Feb 12, 2024). |
| Tanzania, 2014 | Hannah L. Ratcliffe | Ratcliffe HL, Sando D, Lyatuu GW, *et al.* Mitigating disrespect and abuse during childbirth in Tanzania: an exploratory study of the effects of two facility-based interventions in a large public hospital. *Reprod Health* 2016; **13**: 79. |
| Kenya, 2014 | Population Council | Ndwiga C, Warren C, Abuya T, *et al.* Promoting Respectful Maternity Care: A training guide for community-based workshops—Community facilitator’s guide. *Reproductive Health* 2014; published online Jan 1. DOI:10.31899/rh9.1032.  Population Council. Promoting Respectful Maternity Care: A training guide for facility-based workshops—Facilitator’s guide. *Reproductive Health* 2015; published online Jan 1. DOI:10.31899/rh9.1030 |
| Nigeria, 2017 | Federal Ministry of Health | White Ribbon Alliance. Respectful Maternity Care: A Nigeria-focused Health Workers’ Training Guide. Washington DC: Futures Group, Health Policy Project., 2015 https://www.healthpolicyproject.com/pubs/789_WRANToolkitFINAL.pdf (accessed Dec 2, 2024). |
| Afghanistan, 2017 | Federal Ministry of Health | Reproductive, Maternal, Newborn, child and Adolescent Health. Respectful Maternity Care Orientation Package for Health Care Providers: Participants Guide. Afghanistan: Ministry of Public Health, 2017 https://platform.who.int/docs/default-source/mca-documents/policy-documents/operational-guidance/afg-mn-32-01-operationalguidance-2017-eng-523476805-respectful-maternity-care-providers.pdf. |
| Ethiopia, 2017 | Federal Ministry of Health | Compassionate, Respectful and Caring: Health Workforce Training - Participant Manual. Ethiopia: Ministry of Health, Ethiopia, 2017. |
